# Conversion of Racemic Unnatural Amino Acids to Optically Pure Forms by a Coupled Enzymatic Reaction

**DOI:** 10.3390/molecules26051274

**Published:** 2021-02-26

**Authors:** Hannae Lee, Dongchan Kim, Sooin Kim, Hyun Soo Lee

**Affiliations:** Department of Chemistry, Sogang University, 35 Baekbeomro Mapogu, Seoul 121-742, Korea; hannae544@naver.com (H.L.); dongchan94@naver.com (D.K.); sooin27@gmail.com (S.K.)

**Keywords:** genetic code expansion, unnatural amino acids, D-amino acid oxidases, aminotransferases

## Abstract

Genetic code expansion (GCE) technology is a useful tool for the site-specific modification of proteins. An unnatural amino acid (UAA) is one of the essential components of this technique, typically required at high concentration (1 mM or higher) in growth medium. The supply of UAAs is an important limitation to the application of GCE technology, as many UAAs are either expansive or commercially unavailable. In this study, two UAAs in a racemic mixture were converted into optically pure forms using two enzymes, the d-amino acid oxidase (RgDAAO) from *Rhodotorula gracilis* and the aminotransferase (TtAT) from *Thermus thermophilus*. In the coupled enzyme system, RgDAAO oxidizes the d-form of UAAs in a stereospecific manner and produces the corresponding α-keto acids, which are then converted into the l-form of UAAs by TtAT, resulting in the quantitative and stereospecific conversion of racemic UAAs to optically pure forms. The genetic incorporation of the optically pure UAAs into a target protein produced a better protein yield than the same experiments using the racemic mixtures of the UAAs. This method could not only be used for the preparation of optically pure UAAs from racemic mixtures, but also the broad substrate specificity of both enzymes would allow for its expansion to structurally diverse UAAs.

## 1. Introduction

Amino acids are organic molecules containing both amine and carboxylic acid. The most common amino acids are α-amino acids, which are the building blocks of proteins. Proteins consist of twenty canonical amino acids, and their marvelous functions mainly come from the functional groups of the amino acids along with cofactors and post-translational modifications. In addition to their function in proteins, α-amino acids play important roles in the regulation of critical metabolic pathways and cell growth [1,2,3], and their deficiency is associated with diseases in both humans and animals [4,5]. Owing to the crucial roles of amino acids in chemistry and biology, enormous efforts have been made for their synthetic preparation.

One of the important issues in α-amino acid synthesis is the preparation of amino acids in an optically pure form. Except for glycine, all proteinogenic amino acids have at least one stereogenic center, and only the l-form of the amino acids is used for protein synthesis. Optically pure amino acids, including non-proteinogenic amino acids, have been extensively used in the pharmaceutical, nutrition, cosmetic, and agricultural industries [6], prepared by biological fermentation, chemical, and enzymatic synthesis [6,7,8]. Biological fermentation uses biosynthetic systems in microorganisms and enables the cost-effective preparation of proteinogenic amino acids for industrial applications [8]. Chemical synthesis can be applied to diverse, non-proteinogenic amino acids, and has progressed with the development of metal and organic catalysts [7]. Enzymatic synthesis has been extensively used for various amino acids, and enzymes involved in reductive amination, the transamination reactions of keto acids, and ammonia addition to α,β-unsaturated acids have been applied for the preparation of optically pure amino acids [6]. In many cases, systems coupled with another enzymatic reaction for cofactor regeneration or intermediate synthesis have been used. Although these methods have shown their utility in the preparation of various amino acids, their application to diverse non-proteinogenic amino acids is hampered due to limited substrate scope and complications in intermediate synthesis.

As an alternative approach, the deracemization or resolution of racemic mixtures of α-amino acids has been used for the preparation of optically pure α-amino acids [9,10,11,12,13,14,15,16]. Racemic mixtures of α-amino acids are less expensive or synthetically more accessible than their optically pure forms. Both chemical and enzymatic methods have been applied for the deracemization or resolution of racemic α-amino acids. Typically, chemical approaches focus on the separation of each enantiomer by creating a chiral environment in the amino acid itself, or by separating materials such as resins [9,10,11,12]. Enzymatic methods use the stereospecificity of an enzyme for one enantiomer and convert the enantiomer into a chemically differentiable form [13,14,15,16]. When an enzymatic conversion is coupled with another reaction that converts the enzymatic product back into α-amino acids in a racemic form, this cyclic system eventually produces optically pure α-amino acids [13,14,15]. An ideal system for the deracemization or resolution of racemic α-amino acids involves the quantitative and stereospecific conversion of one enantiomer into the other enantiomer. To the best of our knowledge, a coupled enzyme system, in which one enzyme converts one enantiomer in a racemic α-amino acid into an intermediate, and the other enzyme produces another enantiomer from the intermediate in a quantitative and stereospecific manner, has yet to be reported.

The genetic incorporation of unnatural amino acids (UAAs) is a useful technique for the site-specific modification of proteins [17,18,19]. The fact that this technique requires a high concentration (typically 1 mM or higher) of UAAs is one of its key limitations due to the sparse commercial or synthetic accessibility of UAAs. In addition, cells designed for the genetic code expansion (GCE) technique are able to use only the l-form of UAAs; therefore, optically pure UAAs are required for improved efficiency. Unfortunately, a number of the UAAs that have been used for the technique are expensive or commercially unavailable, and their preparation requires multistep chemical synthesis. In addition, some UAAs such as metal-chelating amino acids have cytotoxicity, in which cases an optically pure form is essentially required for better protein yield. Therefore, the development of efficient methods for the synthesis of UAAs in an optically pure form is in high demand. Many efforts have been made to address this issue, including the biosynthesis of UAAs in host cells [20,21], the development of novel synthetic methods [22,23], engineering bacterial uptake systems for UAAs [24], and recycling growth medium containing UAAs [25]. Alternatively, conversion of racemic UAAs into an optically pure form can be a decent solution to address the issue, which has never been applied to GCE technology.

In this study, UAAs in a racemic mixture were converted into their optically pure forms using two enzymes, namely a d-amino acid oxidase (DAAO) and an aminotransferase (AT). DAAO oxidizes the d-form of UAAs and produces the corresponding α-keto acids; then AT converts the α-keto acids into the l-form of UAAs quantitatively and stereospecifically in a coupled reaction system. When applied to a GCE system, the optically pure UAAs obtained using this method were found to produce higher protein yields than the racemic mixtures of the UAAs in the same experiments. Thus, the coupled enzyme system developed in this study represents a useful tool for the quantitative and stereospecific conversion of racemic UAAs into optically pure forms. Furthermore, the broad substrate specificity of the enzymes used in the system allows for the expansion of this approach to structurally diverse UAAs.

## 2. Results and Discussion

### 2.1. Metal-Chelating Amino Acids in GCE Technology

**Figure 1:**
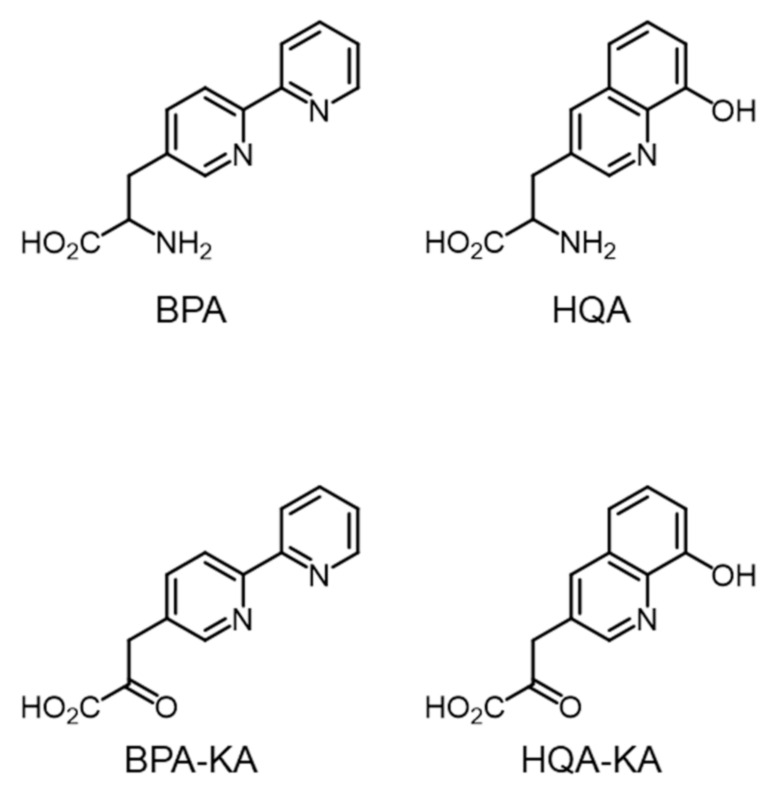
Structures of unnatural amino acids and keto-acids used in this study.

Using the GCE technique, two metal-chelating unnatural amino acids, (2,2′-bipyridin-5-yl)ala- nine (BPA) and (8-hydroxyquinolin-3-yl)alanine (HQA) (Figure 1), were successfully incorporated into the target proteins [26,27,28,29]. The UAAs site-specifically generated a metal-binding site at a defined position in the target proteins. The resulting mutant proteins were utilized for sequence-specific DNA cleavage [27], structure determination by single-wavelength anomalous diffraction (SAD) [28], and metal-affinity protein purification [29]. Although the UAAs produced only mutant proteins in a moderate yield, their metal-binding property caused cytotoxicity at concentrations greater than 1 mM as a result of a decrease in the cellular concentration of metal ions due to their high metal affinity. The protein yield from the genetic incorporation of BPA and HQA is known to decrease at an high (> 1 mM) UAA concentration, such that their toxicity limits the protein yield. Another important issue is that the metal-chelating amino acids are prepared in racemic mixtures [27,28], such that their effective concentration is half of the concentration in cells. In this context, the preparation of BPA and HQA in an optically pure form is in high demand. Thus, we attempted to develop a novel synthetic method for the preparation of optically pure UAAs.

### 2.2. System Design for the Preparation of Optically Pure BPA and HQA

Initially, a transamination reaction was chosen for the target reaction. It was anticipated that α-keto acids BPA–KA and HQA–KA (Figure 1) could be readily converted into BPA and HQA in an optically pure form by an aminotransferase (AT). To this end, AT from *Thermus thermophilus* (TtAT) was selected as the target enzyme. TtAT has been used for similar applications, efficiently converting multiple α-keto acids into UAAs in cells [20]. However, the chemical synthesis of BPA–KA and HQA–KA has failed for unknown reasons.

**Figure 2:**
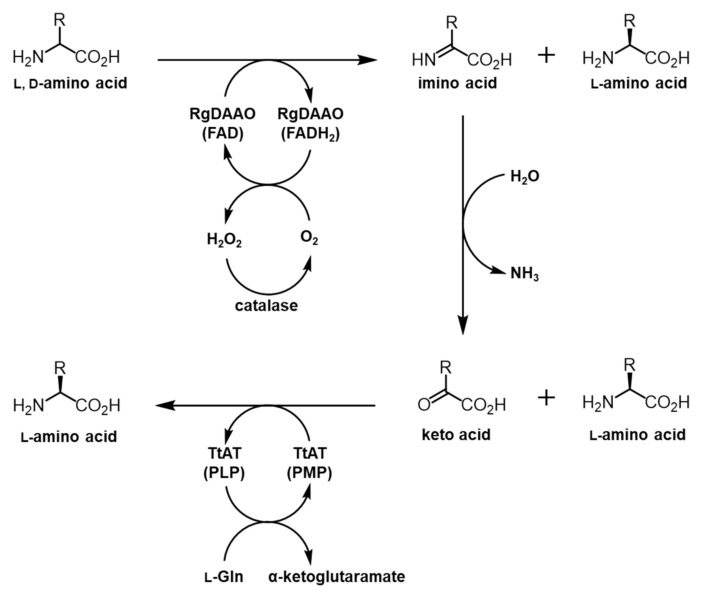
Overall scheme of the coupled enzyme system for conversion of racemic amino acids into their optically pure forms. RgDAAO oxidizes the d-form of a racemic amino acid into the α-keto acid, which is subjected to the transamination by TtAT to produce the l-form of an amino acid. The final product of this process is an optically pure l-form amino acid.

The deracemization of racemic BPA and HQA was considered as an alternative approach. Amino acid oxidases (AAOs) consisting of l-amino acid oxidases (LAAOs) and d-amino acid oxidases (DAAOs) are FAD- or FMN-containing enzymes that oxidize α-amino acids into imino acids, which are converted into α-keto acids by subsequent hydrolysis [30]. AAOs along with amino acid deaminases, which have a similar function as AAOs, have been used for the deracemization of α-amino acids [31,32,33]. To deracemize BPA and HQA, the DAAO from *Rhodotorula gracilis* (RgDAAO) was selected, since this enzyme showed high levels of activity and a broad substrate specificity for bulky amino acids. In a previous report, the enzyme was used to efficiently convert naphthylalanines and naphthyglycines into their oxidized products [33]. Using information from previous studies [20,33,34], a coupled enzyme system was designed to deracemize BPA and HQA, in which RgDAAO oxidizes the d-forms of the UAAs into the α-keto acids and TtAT carries out the transamination reactions to produce the l-forms of the UAAs (Figure 2).

### 2.3. RgDAAO and TtAT Activity for BPA and HQA

**Figure 3:**
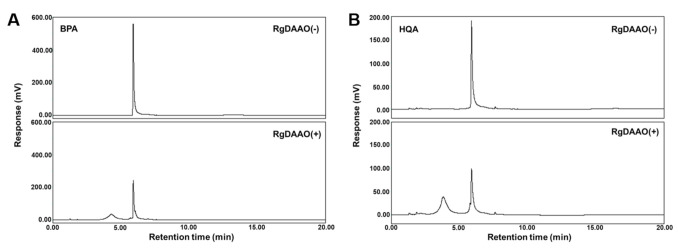
HPLC (C18) analyses of RgDAAO reactions for racemic BPA (**A**) and HQA (**B**). Conditions: 50 mM HEPES-NaOH (pH 8.0), 0.1 M KCl, 1.0 mg/mL catalase, 5 mM UAA, and 5 μM RgDAAO. The reaction mixture was incubated at 25 °C for 15 min. The reaction was quenched by adding methanol, and supernatant from centrifugation was analyzed by HPLC.

RgDAAO was initially tested for its activity toward BPA and HQA. The amino acids were prepared according to methods previously reported for their racemic forms [27,28]. A His_6_-tagged RgDAAO was expressed in *E. coli* BL21(DE3) and purified by affinity chromatography. Since BPA and HQA have not been previously used as substrates for RgDAAO, a peroxidase-coupled enzymatic assay was performed to measure the activity of RgDAAO towards the amino acids [35]. In the assay, RgDAAO oxidized an amino acid substrate and produced an imino acid and hydrogen peroxide. The amount of hydrogen peroxide produced was quantified by a peroxidase that oxidizes *o*-dianisidine, resulting in an absorbance increase at 440 nm. For comparison, BPA, HQA, and d-2-naphthylalanine (d-NPA) were subjected to a peroxidase-coupled enzymatic assay. All produced an absorbance increase at 440 nm, indicating that RgDAAO recognized the UAAs tested as substrates (Figure S1). The Michealis-Menten kinetic parameters were calculated, and RgDAAO showed significant activity for both UAAs, although their *k_cat_/K_m_* values were 6-fold and 11-fold less than that of NPA, respectively (Table 1). The reactions were then analyzed by HPLC. BPA and HQA were treated with RgDAAO, and the reaction products were separated by HPLC. For both UAAs, RgDAAO reduced the amino acid peaks and produced new peaks, with a conversion percentage that was close to quantitative, based on peak integration (Figure 3 and Table 2). The new peaks produced were characterized as the corresponding keto acids by LC-MS (Figure S2). Importantly, the amino acid peaks did not change at half of the initial size with a longer reaction time (up to 60 min; data not shown), which provides strong evidence that the enzymatic reactions by RgDAAO are specific for the d-forms of the UAAs, while their l-forms remained unreacted. This expectation was based on the results of the HPLC analysis and the reported d-amino acid specificity of RgDAAO [31]. Subsequent experiments also further confirmed this expectation (vide infra).

Next, the second enzyme, TtAT, was evaluated for its recognition of BPA-KA and HQA-KA in the transamination reaction. Because both α-keto acids are not commercially or synthetically accessible, the activity of TtAT was indirectly tested using BPA and HQA as amine donors. This indirect assay was based on the fact that good amine donors for TtAT are usually good amine acceptors in their α-keto acid forms [34]. TtAT was incubated with BPA or HQA at varied concentrations in the absence of an amine acceptor, and the conversion of pyridoxal 5’-phosphate (PLP) into pyridoxamine 5’-phosphate (PMP) was measured by UV spectrophotometry. The measurement showed that the absorbance at 430 nm decreased with an increasing concentration of both UAAs (Figure S3). This result indicates that TtAT recognizes BPA and HQA as substrates, although detailed kinetic parameters were not measured because of the inaccessibility of BPA-KA and HQA-KA.

### 2.4. Enzymatic Preparation of Optically Pure BPA and HQA

**Figure 4:**
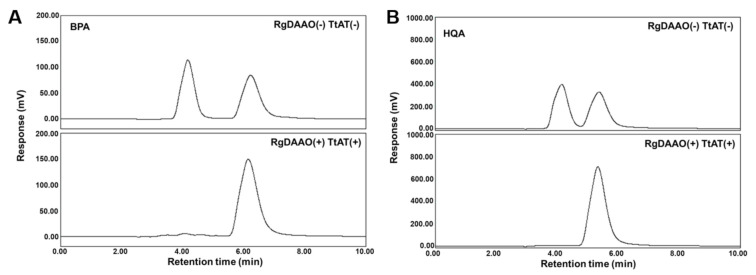
Chiral HPLC analyses of coupled-enzymatic reactions for racemic BPA (**A**) and HQA (**B**). Conditions: 50 mM HEPES-NaOH (pH 8.0), 0.1 M KCl, 1.0 mg/mL catalase, 5 mM UAA, and 5 μM of RgDAAO and TtAT. The reaction mixture was incubated at 25 °C for 30 min. The reaction was quenched by adding methanol. The supernatant obtained after centrifugation was analyzed by HPLC.

Because both RgDAAO and TtAT showed activity for BPA and HQA in enzymatic assays, a coupled assay was designed and tested for the conversion of racemic UAAs into their optically pure forms. Each UAA was initially treated with RgDAAO for 15 min, and TtAT and l-Gln as an amine donor were then added to the reaction mixture. The reaction proceeded for another 15 min, and the mixture was analyzed by chiral HPLC. The control experiments without the enzymes showed two clear peaks for the l- and d-forms of each UAA (Figure 4). In the presence of both enzymes, one of the peaks disappeared and the other peak was enlarged (Figure 4). Based on this result, the left-most peaks for both UAAs with a short retention time were assigned as the d-forms of BPA and HQA. The enlarged peaks were characterized as BPA and HQA by LC-MS (Figure S4). The conversion and enantiomeric excess were greater than or equal to 95% for both UAAs based on the integration of the HPLC traces (Table 2). These results show that the coupled enzyme system efficiently converts the d-forms of BPA and HQA into their l-forms with a high stereospecificity. Although the TtAT reaction is reversible, the reaction proceeded to the desired direction (the direction for l-BPA or l-HQA formation) exclusively in the presence of 10 mM l-Gln (the amine donor).

### 2.5. Application of Optically Pure BPA and HQA to GCE Experiments

**Figure 5:**
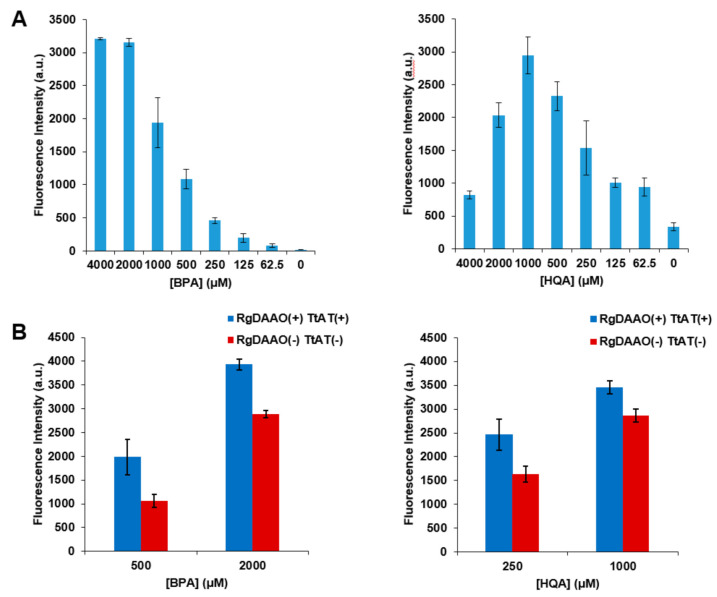
Genetic incorporation of BPA and HQA into EGFP. A GFP gene with an amber stop codon (TAG) at position 39 was expressed in the presence of the aminoacyl-tRNA (aa-tRNA) and aa-tRNA synthetase (aaRS) pair for BPA or HQA. (**A**) The expression was carried out with varied concentrations of racemic BPA or HQA, and GFP fluorescence was measured at 510 nm with excitation at 497 nm. (**B**) The expression was carried out with indicated concentrations of optically pure BPA or HQA prepared from the coupled enzymatic reactions, and GFP fluorescence was measured at 510 nm with excitation at 497 nm.

Next, the UAAs that were enzymatically converted into optically pure forms were subjected to genetic incorporation to evaluate their incorporation efficiency in comparison with that of the racemic forms. A green fluorescent protein (GFP) gene containing an amber codon at position 39 (GFP-Y39TAG) was expressed in the presence of the aminoacyl-tRNA (aa-tRNA) and aa-tRNA synthetase (aaRS) pair for BPA or HQA [27,28]. The GFP-Y39TAG gene was expressed at varied concentrations of racemic BPA or HQA, and their incorporation efficiency was evaluated by measuring GFP fluorescence. The incorporation of BPA or HQA was found to increase with an increasing concentration of the UAAs; however, this increase was saturated or reversed at high concentrations (Figure 5A). This saturation or decrease may have been caused by the toxicity of the UAAs, and this effect was more significant for HQA due to its stronger metal-chelating affinity [29,36]. The GFP-Y39TAG gene was then expressed in the presence of l-BPA or l-HQA produced by the treatment of their racemic forms with the two enzymes. GFP fluorescence was measured at two different concentrations of each l-UAA and compared with those from control samples without the enzymes (Figure 5B and Table 3). The results showed a significant increase in fluorescence, and more importantly, the fluorescence intensities for l-BPA and l-HQA were the same or higher than those for the doubled concentrations of racemic UAAs (Table 3; values in parentheses). In addition, the fluorescence intensities for 2 mM l-BPA and 1 mM l-HQA were significantly higher than the maximum intensities for racemic UAAs, indicating that an increased maximum level of protein expression can be achieved by using l-UAAs. These results were produced as a result of the increase in the effective concentration originating from the high optical purity of the UAAs and the accompanying decrease in the toxicity of the UAAs.

## 3. Materials and Methods

### 3.1. Expression and Purification of RgDAAO

A synthetic gene of RgDAAO with an *N*-terminal His_6_-tag was amplified with primers with NcoI and KpnI restriction sites. The gene was inserted between the NcoI and KpnI sites of pBAD/Myc-His (Invitrogen, Carlsbad, CA, USA) to generate pBAD-RgDAAO, which was then transformed into *E. coli* DH10β; then 5 mL of starter culture was used to inoculate 200 mL of 2×LB medium. The culture was induced at an optical density (OD) of 0.8 (600 nm) by adding arabinose (0.2%, final concentration). The cells were grown at 30 °C for 3 h and harvested by centrifugation. The protein expressed was purified by Ni-NTA affinity chromatography under native conditions, according to the manufacturer’s protocol (Qiagen, Valencia, CA, USA). For enzymatic reactions, the buffer was changed with 50 mM HEPES-NaOH (pH 8.0).

### 3.2. Expression and Purification of TtAT

A C-terminal His_6_-tagged TtAT gene was amplified from *Thermus thermophilus* HB8 genomic DNA with primers with NdeI and BglII restriction sites. The gene was inserted between the NdeI and BamHI sites of pET20b (Invitrogen, Carlsbad, CA, USA) to generate pET20b-ttGlnAT, which was then transformed into *E. coli* BL21 (DE3); then 5 mL of starter culture was used to inoculate 200 mL of LB medium. The culture was induced at an OD of 0.8 (600 nm) by adding IPTG (1 mM, final concentration). The cells were grown at 37 °C for 14–16 h and harvested by centrifugation. The protein expressed was purified by Ni-NTA affinity chromatography under native conditions according to the manufacturer’s protocol (Qiagen, Valencia, CA, USA). For enzymatic reactions, the buffer was changed with 50 mM HEPES-NaOH (pH 8.0)

### 3.3. RgDAAO Reaction

Michaelis-Menten kinetics experiments were carried out by measuring the initial velocities of the conversion of unnatural amino acids into the corresponding imino acids (keto acids). The enzymatic reaction was initiated by adding RgDAAO (11.9 nM for NPA and 119 nM for BPA and HQA) to a solution containing 75 mM phosphate buffer (pH 8.5), 1 mM *o*-dianisidine, 1 U horseradish peroxidase (HRP), and an UAA (indicated concentrations) before incubating at 25 °C. The time-dependent absorbance was measured at 440 nm for each UAA concentration, and the initial velocities were plotted. For HPLC analysis, the reaction was initiated by adding the enzyme (5 μM, final concentration) to a solution containing 50 mM HEPES-NaOH (pH 8.0), 0.1 M KCl, 1.0 mg/mL catalase, and 5 mM BPA or HQA at a total volume of 100 μL. The reaction mixture was incubated at 25 °C for 15 min. The enzyme was precipitated by adding methanol (100 μL), and the supernatant was used for HPLC analysis after centrifugation. For the control, the enzyme was replaced with buffer. Samples were separated by HPLC on a Zorbax Eclipse Plus C18 (4.6 mm × 150 mm, 5 μm; Agilent Technologies, Santa Clara, CA, USA) using a linear gradient elution from water/acetonitrile (95/5) at 2 min to water/acetonitrile (5/95) at 5 min with a constant flow rate (1.0 mL/min). HPLC traces were taken at 254 nm for HQA and 280 nm for BPA. For LC-MS analyses, the same condition was applied with 0.1% TFA in the eluent.

### 3.4. TtAT Reaction

The reaction was initiated by adding the enzyme (20 μM, final concentration) to a solution containing 50 mM HEPES-NaOH (pH 8.0), 0.1 M KCl, and BPA or HQA (indicated concentrations) at a total volume of 200 μL. The reaction mixture was incubated at 25 °C for 5 min, and the absorbance was measured at 430 nm.

### 3.5. Coupled Reaction of RgDAAO and TtAT

RgDAAO (5 μM, final concentration) was added to a solution containing 50 mM HEPES-NaOH (pH 8.0), 0.1 M KCl, 1.0 mg/mL catalase, and 5 mM BPA or HQA at a total volume of 100 μL and incubated at 25 °C for 15 min. TtAT (5 μM, final concentration) and l-Glu (10 mM, final concentration) were then added to the reaction mixture (110 μL, total volume), and the mixture was incubated at 25 °C for 15 min. The enzymes were precipitated by adding methanol (110 μL), and the supernatant was used for HPLC analysis after centrifugation. Samples were separated by HPLC on a CROWNPAK CR-I(+) column (3.0 mmφ × 150 mmL, 5 μm; CHIRAL Technology Korea, Daejeon, Korea) using an isocratic elution with pH 1.0 aqueous perchloric acid/methanol (80/20) and flow rate of 0.3 mL/min. HPLC traces were taken at 254 nm for HQA and 280 nm for BPA. For LC-MS analyses, the condition described in Section 3.3 was used.

### 3.6. Application to the GCE Technique

The GFP gene (emerald GFP) was obtained from a commercial source by gene synthesis, amplified by PCR, and inserted between the BspHI and KpnI sites of pBAD/Myc-His (Invitrogen, Carlsbad, CA, USA) to generate pBAD-emGFP [21]. An amber codon (TAG) was introduced at position 39 in emGFP by site-directed mutagenesis, and the plasmid was co-transformed with pEvol-BPA-RS or pEvol-HQA-RS into *E. coli* DH10β. The cells were then cultured in LB medium supplemented with ampicillin (100 μg/mL) and chloramphenicol (35 μg/mL). The starter culture (200 μL) was transferred to a defined medium (10 mL) (50 mM Na_2_HPO_4_, 50 mM KH_2_PO_4_, 25 mM (NH_4_)_2_SO_4_, 2 mM MgSO_4_, 0.1% (*w*/*v*) trace metals, 0.5% (*w/v*) glycerol, 0.05% (*w/v*) glucose, and 0.36% (*w/v*) amino acids) supplemented with ampicillin (100 μg/mL), chloramphenicol (35 μg/mL), and UAA (indicated concentration) at 37 °C. For BPA or HQA from the enzymatic reactions, the reaction mixtures were added to the culture medium without further processing. Protein expression was induced by adding 0.2% (*w/v*) l-arabinose when the culture optical density (OD 600) reached 0.8, and the culture was grown overnight at 37 °C. The cells (4 × 10^9^) were harvested by centrifugation at 10,000 rpm and 4 °C for 5 min, and the cell pellets were lysed for 1 h with Bugbuster (Novagen, Darmstadt, Germany) (100 μL) supplemented with a benzonase nuclease (100 units) (Sigma-Aldrich, St. Louis, MO, USA). Cell debris was removed by centrifugation at 13,000 rpm and 4 °C for 10 min. The resulting supernatant was used to measure the fluorescence at 510 nm with excitation at 487 nm. This value was calibrated by multiplication with the final cell density (OD_600_).

## 4. Conclusions

The preparation of non-proteinogenic α-amino acids with a high optical purity is a challenging process. Furthermore, in the case that their chirality cannot be acquired from a natural source, such as natural amino acids, their synthesis becomes even harder. Two metal-chelating amino acids used in this study have been applied for the GCE technique to biosynthesize engineered proteins with noble functions. A coupled enzyme system using RgDAAO and TtAT was designed and used to prepare BPA and HQA in an optically pure form. In this system, RgDAAO converted d-amino acids into their α-keto acids, and TtAT transfered an amino group to the keto acids in a stereospecific manner to produce the corresponding l-amino acids. This conversion was almost quantitative for both UAAs, and the resulting amino acids showed 95% or higher enantiomeric excess. After incorporating both optically pure BPA and HQA into GFP, a significantly higher protein expression was observed compared with the same experiment using racemic UAAs. The coupled enzyme system designed in the present study could be applied to other phenylalanine or tyrosine derivatives since the substrate specificity of the enzymes is promiscuous. In addition, further engineering of the enzymes or the use of other homologous enzymes could be used to expand this method to the stereospecific preparation of other UAAs.

## Figures and Tables

**Table 1:** Kinetic parameters of RgDAAO reactions [a].

Substrate	*k_cat_* (min^−1^)	*K_m_* (mM)	*k_cat_/K_m_* (min^−1^mM^−1^)
NPA	1710 ± 85	0.052 ± 0.0087	3.3 × 10^4^
BPA	448 ± 20	0.087 ± 0.010 [b]	0.52 × 10^4^
HQA	124 ± 0.4	0.043 ± 0.0094 [b]	0.29 × 10^4^

[a] Assay conditions: 75 mM phosphate buffer (pH 8.5), 1 mM *o*-dianisidine, 1 U HRP, UAA (varied concentrations), and RgDAAO (11.9 nM for NPA and 119 nM for BPA and HQA) at 25 °C. [b] BPA and HQA were used in racemic forms, and the values were calibrated accordingly.

**Table 2:** Conversion and enantiomeric excesses of the enzymatic reactions.

Substrate	Conversion (%) [a]		Enantiomeric Excess (%)
	RgDAAO Reaction	Coupled Reaction	
BPA	97	95	95
HQA	98	98	98

[a] Based on peak integration of HPLC traces. The numbers are percentages of the theoretical yield (50%).

**Table 3:** Fluorescence intensity of GFP-Y39TAG expressed by the GCE technique using optically pure BPA and HQA prepared by the coupled enzymes, RgDAAO and TtAT.

Samples	GFP Intensity			
	BPA		HQA	
	500 μM	2000 μM	250 μM	1000 μM
l-form [a]	1983	3937	2456	3456
d,l-form [b]	1059 (1942) [c]	2885 (3211) [c]	1633 (2326) [c]	2868 (2036) [c]

[a] Prepared by the coupled enzymes, RgDAAO and TtAT. [b] Prepared by the same condition without the enzymes. [c] EGFP intensity of the doubled concentration of each UAA in racemic form in Figure 5A for comparison. For example, 1942 is the EGFP intensity of 1000 μM BPA in racemic form (Figure 5A).

## Data Availability

The data presented in this study are available on request from the corresponding author.

## Supplementary Materials

The following are available online: Figures S1–S4; Experimental data of enzymatic assays for RgDAAO and TtAT.

## Sample Availability

Samples of the compounds are not available from the authors.
