# Supplementary material for: Conversion of Racemic Unnatural Amino Acids to Optically Pure Forms by a Coupled Enzymatic Reaction
Source: Molecules. 2021 Feb 26;26(5):1274. doi: 10.3390/molecules26051274 (PMC7956486; doi:10.3390/molecules26051274)
Supplement: Supplementary file 1 [file molecules-26-01274-s001.pdf]

*Supplementary Material*

# Conversion of Racemic Unnatural Amino Acids to Optically Pure Forms by a Coupled Enzymatic Reaction and Its Application to Genetic Code Expansion

**Hannae Lee[†], Dongchan Kim[†], Sooin Kim and Hyun Soo Lee[*]**

Department of Chemistry, Sogang University, 35 Baekbeomro Mapogu, Seoul 121-742, Korea; hannae544@naver.com (H.L.); dongchan94@naver.com (D.K.); sooin27@gmail.com (S.K.)

* Correspondence: hslee76@sogang.ac.kr

† These authors contributed equally to this work.

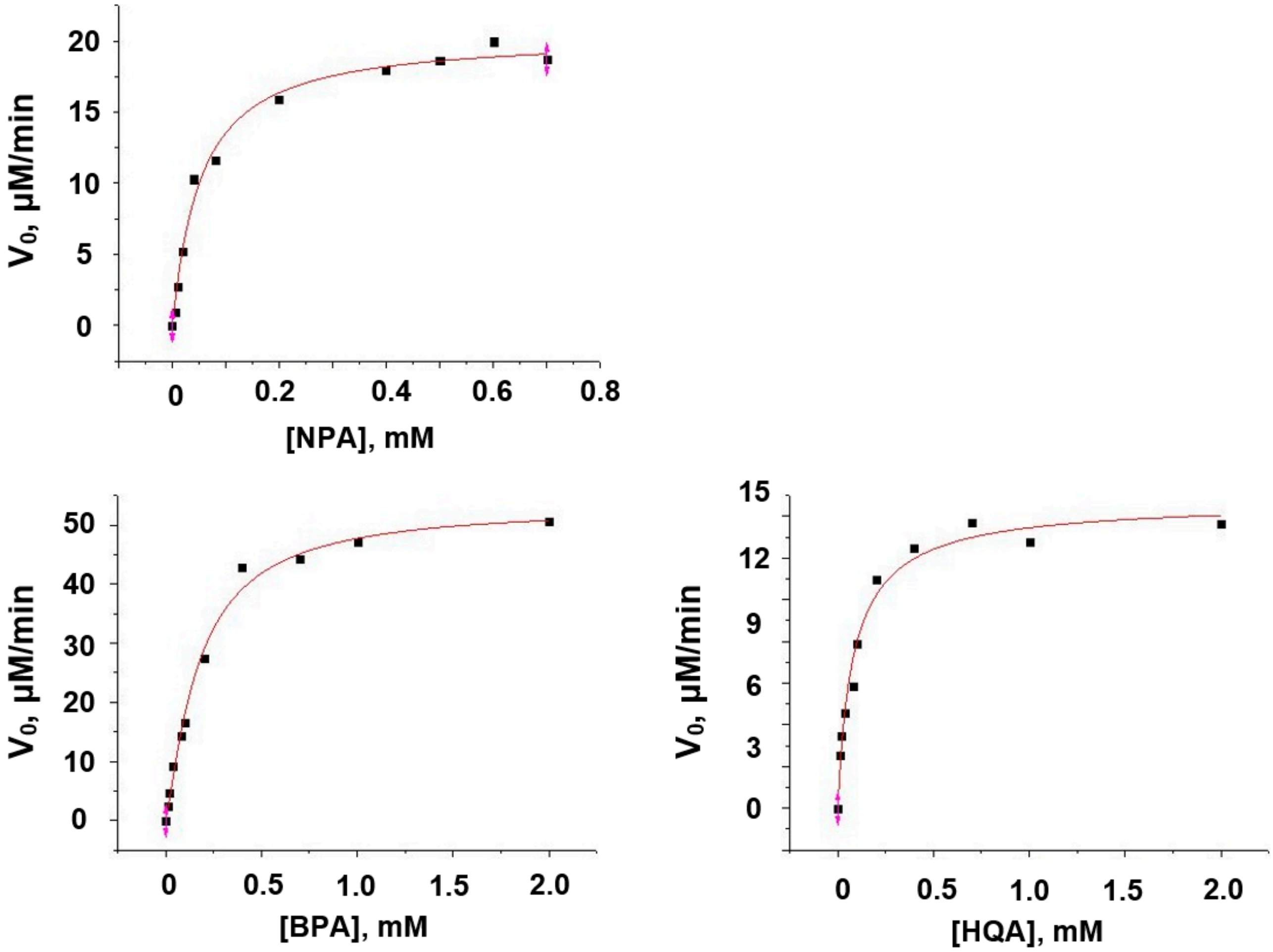


**Figure S1.** Enzyme kinetics of RgDAAO by a peroxidase-coupled enzymatic assay. Conditions: 75 mM phosphate buffer (pH 8.5), 1 mM *o*-dianisidine, 1 U HRP, UAA (indicated concentrations) and RgDAAO (11.9 nM for NPA and 119 nM for BPA and HQA) at 25 °C.

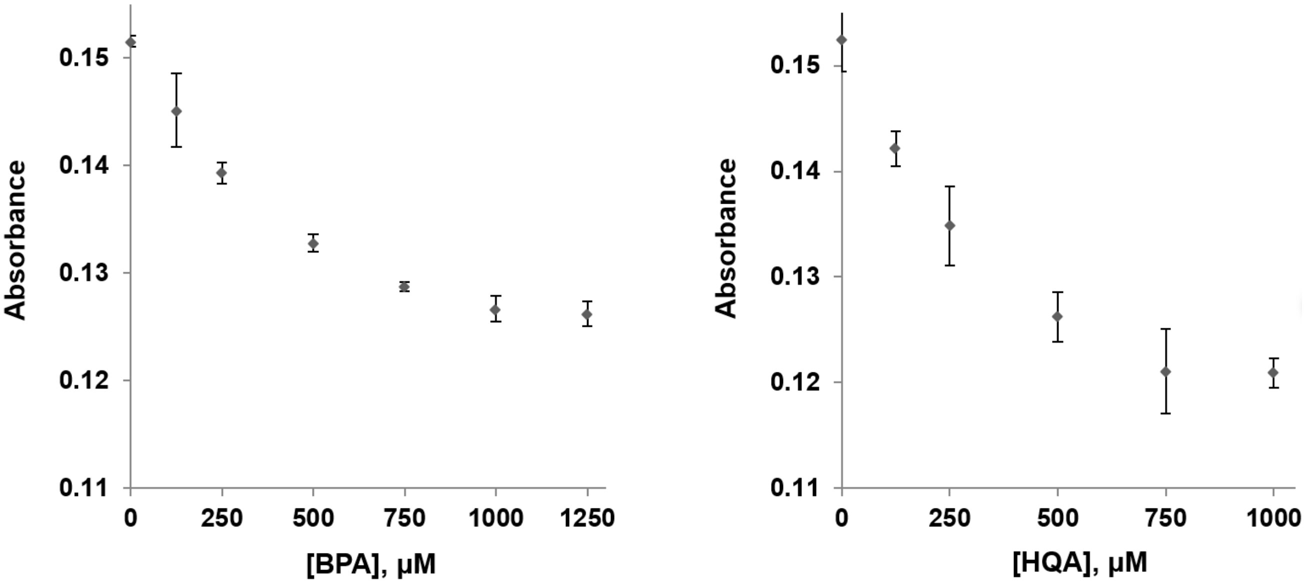


**Figure S2.** TtAT activity for BPA (left) and HQA (right). Conditions: 50 mM HEPES-NaOH (pH 8.0), 0.1 M KCl, UAA (indicated concentrations), and 20 μM TtAT. The reaction mixture was incubated at 25 °C for 5 min, and absorbance was measured at 430 nm.

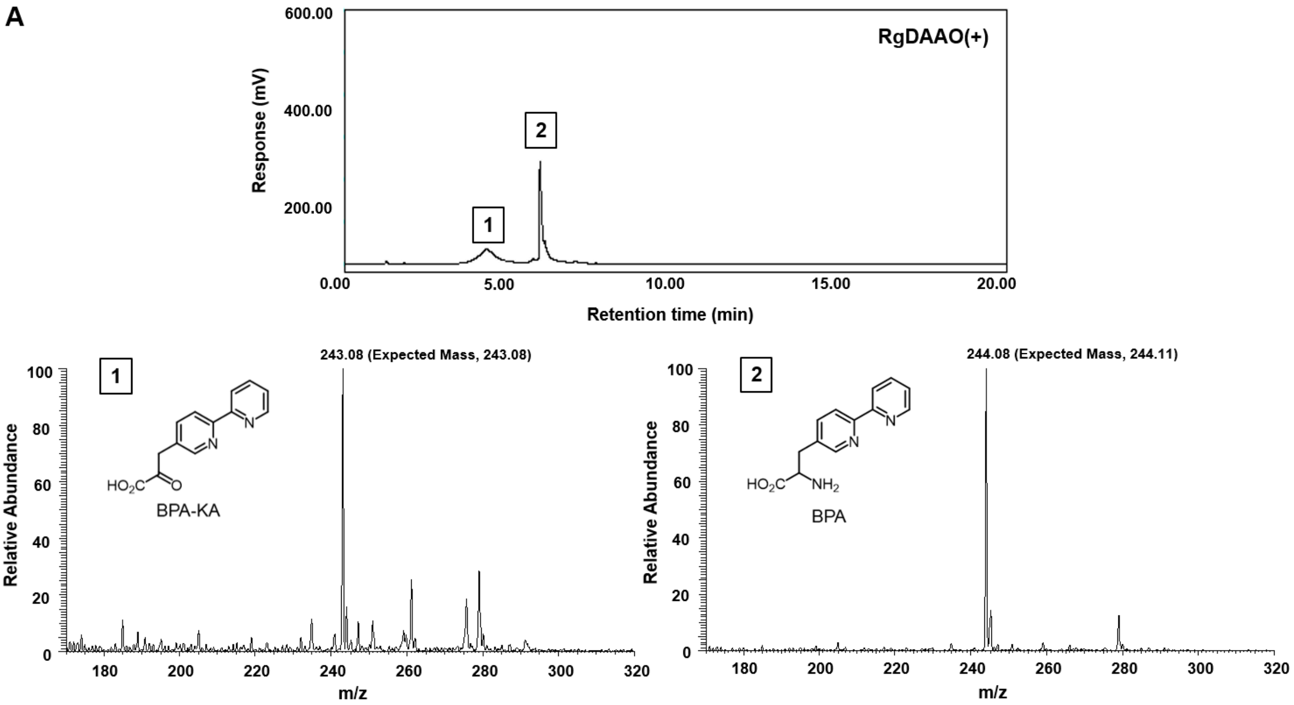


**Figure S3.** LC-MS characterization of the reaction products of racemic BPA (**A**) and HQA (**B**) by RgDAAO. LC traces (280 nm for BPA and 254 nm for HQA) and MS spectra for each peak are shown.

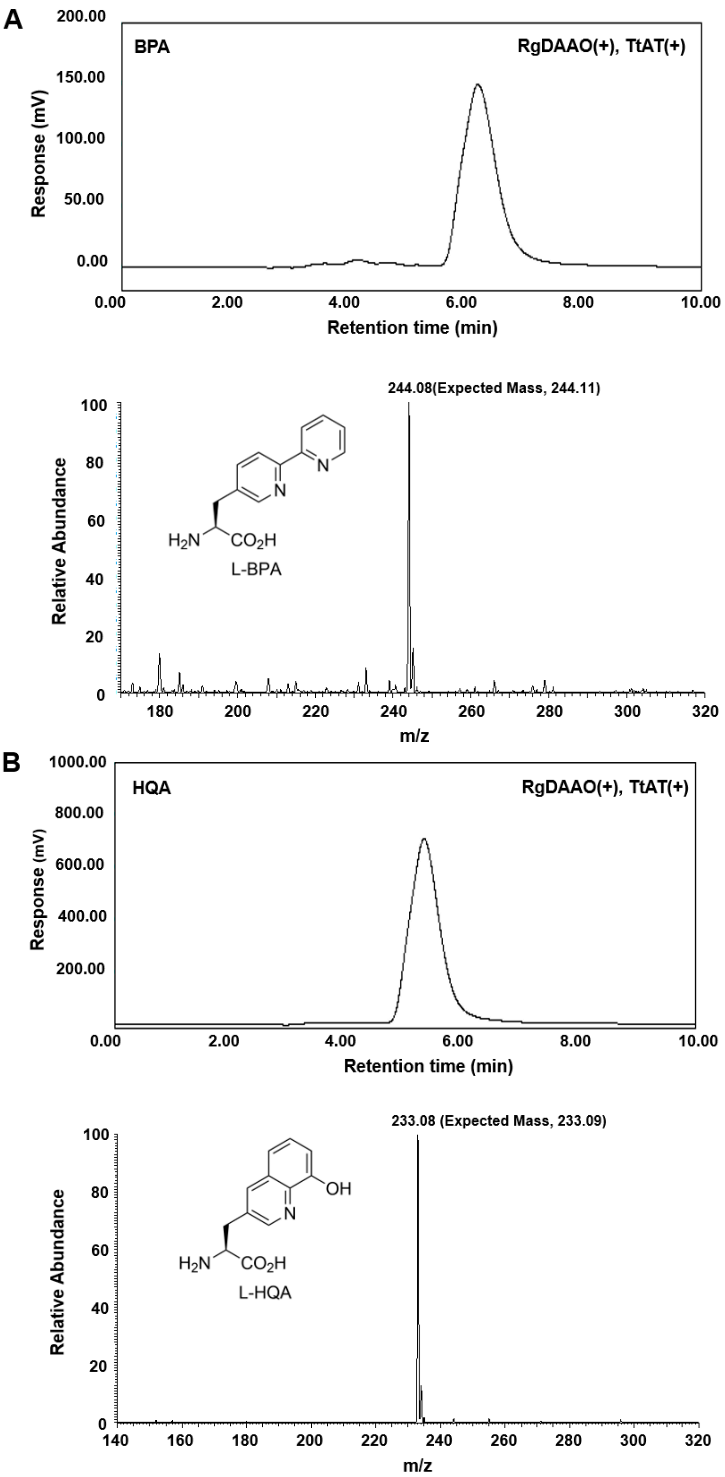


**Figure S4.** LC-MS characterization of the reaction products of racemic BPA (**A**) and HQA (**B**) by the coupled reaction of RgDAAO and TtAT. LC traces (280 nm for BPA and 254 nm for HQA) and MS spectra for each peak are shown.
